# Severity of Oral Mucositis in Children following Chemotherapy and Radiotherapy and Its Implications at a Single Oncology Centre in Durango State, Mexico

**DOI:** 10.1155/2018/3252765

**Published:** 2018-05-10

**Authors:** Ramón G. Carreón-Burciaga, Enrique Castañeda-Castaneira, Rogelio González-González, Nelly Molina-Frechero, Enrique Gaona, Ronell Bologna-Molina

**Affiliations:** ^1^Research Department, Faculty of Dentistry, University of Juarez of Durango State, Durango, DGO, Mexico; ^2^Division of Biological Sciences and Health, Metropolitan Autonomous University, Xochimilco, Ciudad de México, Mexico; ^3^Molecular Pathology Area, School of Dentistry, University of the Republic, Montevideo, Uruguay

## Abstract

**Background:**

Mucositis is an adverse effect of chemotherapy (QT) and/or radiotherapy (RT). The purpose of this study was to investigate the occurrence of oral mucositis in children undergoing cancer treatment.

**Methods:**

Fifty-one children with cancer who had received QT, RT, or both (QT-RT) underwent clinical evaluations; World Health Organization criteria were used to establish the degree and severity of mucositis. The correlations between the clinical data, type of cancer, and therapy were statistically analysed.

**Results:**

Mucositis was present in 88.23% of the patients; 57.78%, 7.78%, and 24.44% received QT, RT, and QT-RT, respectively. Severity scores of 1 and 2 were the most common; scores of 3-4 were observed in patients who received QT-RT or more than 7 treatment cycles. There was a significant association between mucositis, the type of treatment, and the number of cycles received (*p* < 0.05).

**Conclusion:**

It is important to implement therapeutic protocols that help maintain excellent oral health and reduce the risk of oral mucositis. Stomatologists should be consulted to assess patients' oral cavities and provide preventive treatment prior to QT and/or RT administration. It is important to integrate a stomatologist into the oncological working group to focus on preventing and managing oral mucositis.

## 1. Introduction

In Mexico, childhood cancer is the third leading cause of morbidity and mortality among children between 1 and 14 years of age. Such cancers include myeloproliferative diseases (i.e., lymphoma and leukaemia) and tumours of the central nervous system (CNS) [1, 2]. Chemotherapy (QT) and radiotherapy (RT) are commonly used to treat these conditions. QT involves the use of chemicals or drugs that destroy or prevent the reproduction of cancer cells. RT uses radioparticles or high-energy waves (such as gamma rays, electrons, or protons) to damage or destroy cancer cells that are radiosensitive [2].

The adverse effects of QT and RT are related to their cytotoxic activities against noncancerous cells of the body [2, 3], resulting in anatomical and functional conditions such as dysphagia, vomiting, diarrhoea, malnutrition, arthralgia, exanthem, cardiac toxicity, renal toxicity, alopecia, haemorrhage, anaemia, and myelosuppression [4]. The oral cavity is also frequently affected, as lesions can appear which aggravate existing pathologies; such lesions also develop as a consequence of the systemic adverse effects of QT and the local effects of RT on the head and neck [5]. A variety of oral lesions that result from cancer treatment have previously been described, including mucositis, xerostomia, tooth decay, dysgeusia, trismus, mucosal ulcerations, sores, gingival bleeding, periodontitis, viral infections, bacterial infections, fungal infections, and necrosis [6–8]. A significant percentage of oral lesions that are caused by anticancer treatments may be reversible, while others produce permanent sequelae.

Mucositis is a toxic inflammatory reaction that affects the entire gastrointestinal tract, although it is most consequential at the oropharyngeal level. It can affect the patient's general condition and potentially limit the ability to tolerate food and intake fluid. The lesions associated with mucositis are mainly located in the nonkeratinized oral mucosa [7, 8].

QT and RT cause inflammation and ulceration through tissue damage resulting from a sequence of chemical, metabolic, and biological events that occur in several stages. The onset phase occurs following the formation of free radicals [8, 9]. These free radicals produce cellular damage that in turn causes the activation of proinflammatory transcription factors such as nuclear factor-*κ*B that triggers the increased production of inflammatory cytokines. Such inflammation marks the beginning of the ulceration phase that is influenced by the action of cytokines as well as the patient's immune system status and oral bacterial flora [10]. The primary cytokines involved are interleukin- (IL-) 1, IL-6, and tumour necrosis factor-*α*. Ulceration and infection are followed by the healing phase that involves reepithelialization of the damaged tissue.

Mucositis, regardless of grade, can be controlled or cured either with treatment or by the withdrawal of QT/RT, as new cell proliferation, immunological recovery, and adequate control of the bacterial flora ameliorate the condition [11–13].

Mucositis can be measured clinically through toxicity scales such as general mucositis assessment scales, multivariable mucositis scales, and treatment-specific scales. Of frequent use is the World Health Organization (WHO) scale, which classifies the severity of lesions into 4 grades, with grade 0 exhibiting no symptoms while grade 4 indicating the highest severity that renders feeding impossible and requires a catheter or parenteral feeding [14].

The purpose of this study was to evaluate the incidence and severity of oral mucositis at an oncological centre in Durango state, Mexico, and to study the correlation between oral mucositis and the type of oncological treatment received as well as and the frequency of QT/RT sessions or cycles undergone by paediatric patients.

## 2. Methods

We performed an observational, longitudinal clinical study on a paediatric cancer patient population comprising both sexes. We evaluated preschoolers and schoolchildren diagnosed with acute lymphoblastic leukaemia, (CNS) tumours, and lymphoma who were hospitalized or received chemotherapy and/or radiotherapy at the Durango State Cancer Centre between January 2016 and November 2016. Patients receiving antineoplastic treatment with QT and/or RT of the head and neck were included in the study; patients who received bone marrow transplantation, or other types of therapies such as hormone therapy and immunotherapy, and those who underwent tumour surgery without requiring QT and/or RT were excluded.

QT and RT treatments were administered in cycles; a single cycle involved the administration of QT agents and/or an external RT session followed by a rest period.

QT was used for all patients with stage IV acute leukaemia and lymphoma. RT was used for patients with CNS tumours and stages IA and IIA lymphoma. QT and RT in combination were used in patients with leukaemia who required CNS prophylaxis and in those with stage III lymphoma.

To evaluate the severity of mucositis, the WHO criteria of 1979 (that are still in effect to date) were followed. These criteria classify mucositis and its severity into 4 grade levels and 4 phases [14]. Each phase is interdependent and is linked to the effects of QT or RT on the epithelium of the mouth and the action of cytokines, as well as on the state of the patient's immune system and oral bacterial flora [10, 13]. Mucositis grades are as follows.

*Mucositis*

*Grade 0.* This stage includes absence of mucositis.

*Grade 1* (inflammatory, initiatory, or vascular). In this acute phase, IL-1 and tumour necrosis factor-*α* are released. This phase is characterized by erythema and generalized oedema of the mucosa, but no pain.

*Grade 2* (epithelial or amplification of signals). In this stage, deep ulcerative lesions occur owing to the release of cytotoxic agents 4-5 days after the initiation of treatment. These ulcerative lesions are not extensive and cause slight pain; the swallowing of solids is still possible.

*Grade 3* (ulcerative and bacteriological). Following the loss of defense barriers, this stage (which appears 12–14 days after initiating treatment) has a noticeable negative effect on the general condition of the patient and presents a risk of infection. The ulcers are extensive, the gums are markedly oedematous, and the saliva is very thick; there is moderate pain and only liquids can be swallowed.

*Grade 4.* Ulcers are more extensive, bleeding gums and infection are observed, saliva is absent, pain is very intense, and discomfort prevents the patient from ingesting solids and liquids (Table 1).

Data were recorded using Microsoft Excel and later processed using the SPSS for Windows (version 21) statistical package (IBM Corp, Armonk, NY) for the calculation of absolute frequencies, relative frequencies, measures of central tendency, and dispersion. Chi-square test was used for expected values and the Kruskal-Wallis test was applied to detect the differences between severity of mucositis and grade grouped with the independent variables. The results were tabulated and the data analysed with Microsoft Excel and later processed using SPSS statistical package (SPSS 21, IBM Corp, New York, USA). Differences were considered significant at *p* ≤ 0.05.

## 3. Results

We analysed 51 patients, of whom 30 (58.82%) were male and 21 (41.18%) were female; 35 patients (68.63%) were 2–5 years old while 16 (31.37%) were 6–12 years old. Thirty patients (58.8%) had acute lymphoblastic leukaemia, 12 (23.5%) had lymphoma, and 9 (17.6%) had malignant CNS tumours.

**Figure 1:**
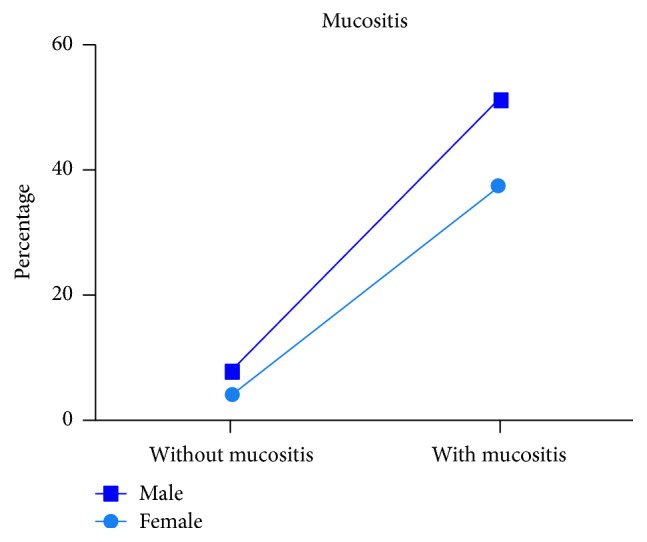
Distribution of patients with and without mucositis according to gender, expressed as percentage of patients.

Forty percent of the patients who received QT reported mucositis symptoms in the first week after starting the first cycle of treatment; at 2 weeks, the majority had either developed mucositis or experienced a worsening of symptoms. Patients with mucositis associated with RT (with or without QT) developed oral lesions between the second and third weeks. The size of grades 2 and 3 ulcers ranged from 0.5 to 2 cm, while grades 3 and 4 ulcers reached 3-4 cm in diameter. Ultimately, 45 of the 51 patients (88.24%) developed mucositis (Figure 1).

In terms of the severity of mucositis, the majority of patients presented with grade 1 or 2, while only 22.2% presented with grade 3 or 4.  Table 2 shows the degrees of mucositis found in the affected population according to the WHO scale relative to sex and age. The 6 patients who did not have mucositis included 2 girls and 4 boys; 2 were in the 2–5-year age group (preschoolers) and 4 were in the 6–12-year age group (schoolchildren). The highest frequency and severity of mucositis occurred in the 2–5-year age group.  Table 3 shows the degree of mucositis relative to cancer type and treatment with QT and/or RT. A total of 55.56% of patients with leukaemia had grade 1 or 2 mucositis, while 6.67% had grade 3. In patients with lymphoma, 13.33% had grade 1 or 2 mucositis, while 4.44% had grade 3. In patients with CNS tumours, 8.89% had grade 1 or 2 mucositis and 11.11% had grade 3 or 4.

Most patients with mucositis received QT (57.78%); however, all patients who underwent both QT and RT developed mucositis. Moreover, patients who received both QT and RT had the highest severity of the condition. Of the six patients who did not develop mucositis, 4 received QT and 2 received RT (Table 3). A higher number of treatment cycles was significantly associated with a greater frequency and severity of mucositis (*p* ≤ 0.05).

## 4. Discussion

A high prevalence of mucositis was found in paediatric patients receiving QT and/or RT. Our observed incidence rate of 88.23% was greater than those among adults reported in the literature [14, 15] but similar to that found in a study of Mexican children with lymphoblastic leukaemia undergoing treatment with QT (81.6%) [15]. The incidence and severity of mucositis were markedly higher in children under 6 years of age (73.33%), likely because the oral cavity, including the mucosa, is still developing in children of this age group. Most children who developed mucositis had acute lymphoblastic leukaemia (63.82%), which was consistent with a previous study by Gordón-Núñez et al. [15]. Mucositis was less common in children with malignant lymphomas and CNS tumours [1–3]. Although mucositis appeared to occur more in boys than in girls, sex was not a statistically significant factor in the development of mucositis after adjusting for sex.

Most patients had grades 1 and 2 mucositis (77.78%), while grades 3 and 4 were only present in 22.22% of the study population. When correlated with cancer type, grades 3 and 4 were found to have a high association with CNS tumours owing to the nature of treatment, which invariably requires QT combined with RT of the head and neck; these additive treatments cause increased toxicity. The most commonly used treatment was QT (58.8%), followed by a combination of QT and RT to the head and neck (23.5%) and RT (17.7%); these treatments were correlated with the types of cancers being treated. Patients who only received RT only developed grades 1 and 2 mucositis.

As for the number of cycles of QT and/or RT, mucositis began to appear after the first treatment cycles were administered, and the percentage of patients with mucositis, as well as its severity, significantly increased as treatment progressed, and more cycles were administered [16–18].

Oral health remains a public health concern in Mexico, including in Durango state where 50% of children have dental problems (especially caries); hence, it is possible that the development of oral mucositis is related to poor oral hygiene [19]. It is therefore important to implement preventive and therapeutic guidelines to maintain excellent oral health and reduce the risk of developing oral mucositis. Ávila-Sánchez et al. [19] observed a lower prevalence of oral mucositis than that reported in the global literature after implementing a dental health protocol; the severity of oral mucositis was reduced within 7 days of implementing this protocol. Therefore, we also plan to introduce an oral health protocol at our institute to reduce the risk of oral mucositis. Prior to the administration of QT and/or RT, a thorough oral examination should be performed to eliminate risk factors that cause mucosal irritation and infection with the aim of preventing and reducing oral lesions. This monitoring and treatment should persist throughout all sessions [20]. Napenas et al. [21] recommend that patients visit a stomatologist to receive preventive treatment prior to commencing anticancer therapy and that these patients should continue receiving such therapies during treatment. Moreover, they recommend that any mucositis that develops should be directly treated to alleviate symptoms and eliminate oral infections.

## 5. Conclusions

We found a significant correlation between the type of cancer therapy administered as well as the number of treatment cycles received and the severity of mucositis. It is important to note that mucositis is a high-incidence complication during oncologic therapies; therefore, close observation to reduce or prevent the risk of this complication should be maintained.

Multidisciplinary patient management, in which a stomatologist joins the working group to monitor oral appearance before, during, and after QT and RT are administered, is recommended. In our country, the principal problem of the participation of stomatologist to joining to oncological team is the absence of adequate training in oncological care; however the oncology institutes have specialized training courses for stomatologist in the care of cancer patients, focused on care of the oral complications due to oncological treatment. The participation of a stomatologist to help maintain an excellent oral hygiene is important to prevent oral mucositis or to reduce its severity and/or symptoms should it occur. Diagnostic and therapeutic protocols and algorithms related to complications that arise from anticancer therapy, including mucositis, should be used. These procedures can minimize aggravation to the digestive mucosa and include preventive measures to avoid the development or worsening of mucositis; this helps ensure that cancer patients have fewer oral complications and less pain. This will also allow them to ingest an adequate diet that, in conjunction with cancer treatment, contributes to their physical and psychosocial well-being, a prompt recovery, and ultimately an improved prognosis.

## Figures and Tables

**Table 1:** The World Health Organization grades of oral mucositis [14].

Grade	Description
0	No changes
1	Soreness/erythema
2	Soreness/erythema + ulceration + ability to eat solid foods
3	Soreness/erythema + ulceration + ability to use a liquid diet only
4	Soreness/erythema + ulceration + no possible oral alimentation

**Table 2:** Patients according to the degree of mucositis, grouped by severity of mucositis according to gender and age groups, expressed as number of patients.

Degree of mucositis severity									
	Severity of mucositis								
	0	1	2	3	4	*p* value	1-2	3-4	*p* value
Gender									
Male	4	10	8	6	2	*≤0.001* ^*∗*^	18	8	*≤0.001* ^*∗*^
Female	2	12	5	2	-* *-		17	2	
*Total*	*6*	*22*	*13*	*8*	*2*		*35*	*10*	
Age group									
2 to 5 years	2	16	9	6	2	*≤0.001* ^*∗∗*^	25	8	*≤0.001* ^*∗∗*^
6 to 12 years	4	6	4	2	-* *-		10	2	
*Total*	*6*	*22*	*13*	*8*	*2*		*35*	*10*	

^*∗*^ *p* < 0.01, gender and severity of mucositis; *X*^2^: 23.216. ^*∗∗*^*p* < 0.01, age group and severity of mucositis; *X*^2^: 29.059.

**Table 3:** Patients according to the degree of mucositis, grouped by severity of mucositis according to the type of cancer, treatment, and number of cycles, expressed as number of patients.

Degree of mucositis severity									
	0	1	2	3	4	*p* value	1-2	3-4	*p* value
Type of cancer									
Leukaemia	2	17	8	3	0	*0.001* ^*∗*^	25	3	*0.019* ^†^
Lymphoma	4	5	1	2	0		6	2	
SNC Tumour	0	0	4	3	2		4	5	
*Total*	*6*	*22*	*13*	*8*	*4*		*35*	*10*	
Treatment									
Chemotherapy	4	16	8	2	0	*0.001* ^*∗*^	24	2	*≤0.001* ^*∗*^
Radiotherapy	2	5	3	0	0		8	0	
Chemo-Radio	0	1	2	6	2		3	8	
*Total*	*6*	*22*	*13*	*8*	*2*		*35*	*10*	
Cycles									
1–6	5	17	5	1	0	*0.026* ^†^	22	1	*0.003* ^*∗*^
>6	1	5	8	7	2		13	9	
*Total*	*6*	*22*	*13*	*8*	*2*		*35*	*10*	

^*∗*^ *p* ≤ 0.01;^†^*p* ≤ 0.05. Kruskal-Wallis test.
